# Effects of Facial Expression and Facial Gender on Judgment of Trustworthiness: The Modulating Effect of Cooperative and Competitive Settings

**DOI:** 10.3389/fpsyg.2018.02022

**Published:** 2018-10-22

**Authors:** Yan Dong, Yi Liu, Yanfei Jia, Yongna Li, Chen Li

**Affiliations:** Department of Psychology, Renmin University of China, Beijing, China

**Keywords:** facial expression, facial gender, cooperative setting, competitive setting, trustworthiness judgments

## Abstract

People often judge trustworthiness based on others’ faces (e.g., facial expression and facial gender). However, it is unclear whether social context plays a moderating role in forming trustworthiness judgments. Based on the emotions as social information (EASI) model, differing contexts may impact the effect of facial expression; however, there is no evidence demonstrating that differing contexts will or will not influence the effect of facial gender. In this study, we used two experiments to examine how facial expression and facial gender affect facial trustworthiness judgments and whether the effects on facial trustworthiness judgments are consistent in cooperative and competitive settings. Twenty-seven undergraduates (14 female; *M*_age_ = 21.81 years, *SD* = 2.66) participated in experiment 1. The results showed significant main effects of facial expression and facial gender as well as the interaction between them. To examine the social context effect, 28 undergraduates (18 female; *M*_age_ = 20.93 years, *SD* = 2.94) participated in experiment 2. The results showed the main effects of facial expression, facial gender, and social context. Moreover, there was a significant interaction between facial gender and facial expression and a marginally significant interaction between social context and facial expression. These results suggest that in the process of judging facial trustworthiness, individuals’ judgments are affected by both facial expression and facial gender. Furthermore, the effect of facial gender on facial trustworthiness judgments presents cross-situational stability, and the role of facial expression is influenced by the settings. These findings support and expand the EASI model.

## Introduction

Trustworthiness judgments can be made at first glance. Individuals usually make trustworthiness judgments of strangers based on facial cues, such us facial expression and facial gender (Atkinson et al., 2005; Buchan et al., 2008; Aguado et al., 2009; Todorov et al., 2009; Willis and Todorov, 2010; Dong et al., 2014). Some researchers explored the relationship between facial expression and trustworthiness judgments of faces. Based on different paradigms, researchers found that perceptions of trustworthiness and facial expression were highly related (Oosterhof and Todorov, 2008, 2009; Said et al., 2009; Dong et al., 2014). Other previous studies discussed the effect of facial gender on trustworthiness judgments (Buchan et al., 2008), found that female faces were usually perceived as more trustworthy than male faces. It remains unclear, however, whether the processing of these two cues occurs independently (Haxby et al., 2000, 2002), or is simultaneously interactive (Hess et al., 2000; Atkinson et al., 2005; Aguado et al., 2009; Ozono et al., 2010; Dong et al., 2014; Hack, 2014).

The effects of these two cues, however, may be different in different settings. According to the emotions as social information (EASI) model (Van Kleef et al., 2004, 2010a; Van Kleef, 2009), social context can affect behavior by regulating the way we process emotional information. Other people’s emotional states can affect the observer’s behavior by triggering the observer’s inference process and emotional response, and the observer’s epistemic motivations and the situational social context play a role in regulating this effect. In light of EASI, in cooperative settings, individuals are more likely to use an emotional response to process the emotional information, while in competitive settings, individuals may be more likely to use the inference processing pathway to process the emotional information of others. This suggests that the influence of others’ emotions on our judgments may depend on the specific social context. Until now, no research has illustrated whether facial gender’s influence on trustworthiness judgments can be affected by cooperative and competitive settings. Male face, however, may be more aggressive in competitive settings, leading to decreased trustworthiness; though the same aggressive male face may be perceived as more competent in cooperative settings, leading to increased trustworthiness.

Due to the limited research on the effect of facial expression and facial gender on facial trustworthiness judgment using rapid facial trustworthiness judgment task, we first conducted Experiment 1 to ensure that the paradigm was reasonable and to test the independent or simultaneous effect of facial expression and facial gender. In Experiment 2, we manipulated the social context on the basis of Experiment 1 to test if the effect of facial expression and facial gender would be influenced by context. Based on previous literature (e.g., Hack, 2014), we hypothesized that the effect of the cues were simultaneously interactive during trust judgments, and the effect of facial expression would be susceptible to social context, while the effect of facial gender would be stable in different settings.

## Materials and Methods

### Participants

The prior analysis showed that there should be 24 and 19 participants in Experiment 1 and 2 in order to reach a power of 0.8. Thus, in Experiment 1, there were 27 undergraduates (14 female; *M*_age_ = 21.81 years, *SD* = 2.66), and in Experiment 2, there were 28 undergraduates (18 female; *M*_age_ = 20.93 years, *SD* = 2.94). All participants came from a Chinese university.

### Procedures

In both experiments, participants were asked to finish a facial trustworthiness judgments task. For each trial, a fixation point “+” appeared in the center of the screen and remained for 1000 ms, and was followed by a face picture. The face stimuli were selected from the Chinese Facial Expression Picture System (Gong et al., 2011). We randomly selected 180 emotional face pictures from the database (3 facial expressions (happy, neutral, angry) × 2 facial gender × 30 pictures), although the trustworthiness ratings of those faces were not measured before the study, we unified several characteristics (e.g., skin color, hair, and mole). Participants were asked to judge the trustworthiness of the face by pressing one of the keys 1 (completely untrustworthy) –7 (completely trustworthy) in the upper left corner of the keyboard. Experiment 1 consisted of a total of 180 trails. The face stimuli were presented in random order. In Experiment 2, participants had to imagine they were in a specific situation when reading a description of a cooperative and a competitive setting (He, 2013) before the task. The presentation order of the two settings was counterbalanced between participants. Each participant should accomplish the task in Experiment 1 twice. There was a 10-min break after completing the first task.

## Results

In Experiment 1, a three (facial expression: angry, neutral, happy) × two (facial gender: female, male) ANOVA was conducted (Table 1). There were significant main effects of facial expression (*F*(2,52) = 104.72, *p* < 0.001, η^2^ = 0.80) and facial gender (*F*(1,26) = 50.93, *p* < 0.001, η^2^ = 0.66). The *post hoc* analysis of facial expression showed that people perceived happy faces to be the most trustworthy, followed by the neutral face, rating angry faces as the most untrustworthy (*p*s ≤ 0.001). Moreover, people rated female faces more trustworthy than male faces. There was a significant interaction between facial expression and facial gender. A simple test showed that the effect of facial gender was significant for all expressions (*F*s ≥ 17.22, *p*s < 0.001). Follow-up independent samples *t*-tests revealed that people perceived female faces as more trustworthy than male faces (*t*s ≥ 4.15, *p*s < 0.001).

**Table 1:** Descriptive statistics for the trustworthiness ratings of male and female faces with different expressions in Experiment 1 and 2, the numbers in the parentheses under the mean value are the min and max values the participants rated in each condition.

			Happy faces		Neutral face		Angry faces	
			*M* (Min–Max)	*SD*	*M* (Min–Max)	*SD*	*M* (Min–Max)	*SD*
Experiment 1		Male faces	4.16 (2.17–5.77)	0.92	3.69 (2.20–4.77)	0.69	2.59 (1.23–4.03)	0.65
		Female faces	4.76 (3.30–5.67)	0.61	4.12 (2.97–5.20)	0.57	2.81 (1.83–3.60)	0.50
Experiment 2	Competitive setting	Male faces	3.98 (1.80–5.87)	1.03	3.49 (1.87–5.07)	0.87	2.70 (1.07–4.27)	0.90
		Female faces	4.55 (2.73–6.07)	0.97	3.95 (2.27–5.38)	0.84	2.79 (1.20–4.20)	0.86
	Cooperative setting	Male faces	4.59 (2.07–6.33)	1.08	3.97 (1.27–5.40)	1.00	2.90 (1.00–4.67)	1.01
		Female faces	5.08 (2.73–6.67)	0.90	4.51 (2.13–5.80)	0.83	2.92 (1.00–4.47)	0.87

**FIGURE 1:**
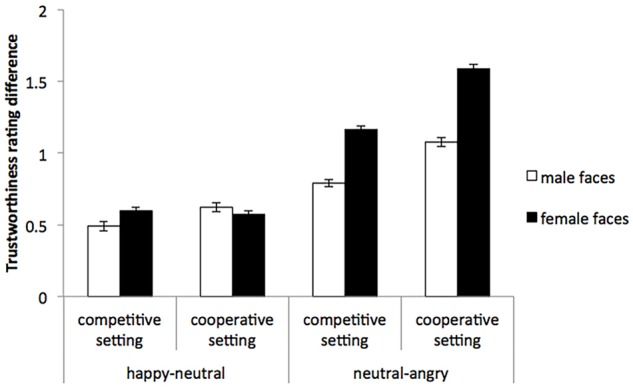
The mean differences between trustworthiness ratings of the neutral and emotional faces in different genders in Experiment 2.

In Experiment 2, we used the mean difference between ratings of neutral faces and emotional faces (neutral faces minus angry faces or happy faces minus neutral faces) as our measure of facial expression effect (descriptive data shown in Table 1; Caulfield et al., 2016). Thus, a two (facial expression: angry, happy) × two (facial gender: female, male) × two (condition: cooperative, competitive) ANOVA was conducted (Figure 1). The result revealed significant main effects of facial expression, *F*(1,27) = 9.68, *p* = 0.004, η^2^ = 0.26, facial gender, *F*(1,27) = 23.56, *p* < 0.001, η^2^ = 0.47, and condition, *F*(1,27) = 5.29, *p* = 0.029, η^2^ = 0.16. Specifically, the angry faces effect was larger than the happy faces effect, and the facial expression effects were larger in female faces and cooperative condition than in male faces and competitive condition. These effects were qualified by a significant interaction between facial gender and facial expression, *F*(1,27) = 7.56, *p* = 0.011, η^2^ = 0.22, and a marginally significant interaction between condition and facial expression, *F*(1,27) = 2.99, *p* = 0.095, η^2^ = 0.10. Simple tests of the interaction between facial gender and facial expression showed a marginally significant effect of facial expression for the male faces, *F*(1,27) = 3.63, *p* = 0.068, and a significant effect for the female faces, *F*(1,27) = 14.62, *p* = 0.001. Follow-up independent sample *t*-tests revealed that, for male faces, the angry faces effect was marginally larger than happy faces effect, *t*(54) = 1.86, *p* = 0.068, and, for female faces, the angry faces effect was also larger than the happy faces effect, *t*(54) = 4.24, *p* < 0.001. Simple tests of the interaction between condition and facial expression showed a significant effect of condition on the angry faces effect, *F*(1,27) = 7.96, *p* = 0.009, but no significant effect of condition on the happy faces effect, *F*(1,27) = 0.19, *p* = 0.665. Follow-up independent samples *t*-tests revealed that, the angry faces effect was larger under the cooperative condition than the competitive condition, *t*(54) = 2.82, *p* = 0.009.

## Discussion

Experiment 1 revealed the simultaneous effect of facial expression and facial gender on trustworthiness judgment. Experiment 2 found that social settings modulated the effect of facial expression on trustworthiness—specifically, the facial expression effect on trustworthiness judgments triggered by angry faces was larger in the cooperative setting than in the competitive setting.

This study revealed that facial expression and facial gender can both be used as cues for trustworthiness judgments, among which happy faces and female faces were more likely to be evaluated as worthy of trust. These results were consistent with previous studies (Keller, 2001; Said et al., 2009; Riedl and Javor, 2012; Dong et al., 2014). Anger expression generally communicates negativity, i.e., refusals and warnings to stay away, happy expression generally denotes approachable, safe, and reliable situations (Heerdink et al., 2014). Besides, females are generally considered to be weaker and more reliable than males (Williams, 2001). We also found that the low trustworthiness caused by the angry faces has a greater damage effect compared to the promotion effect elicited by the happy faces. Similarly, Rule et al. (2012) found that compared with trustworthy faces, untrustworthy faces were remembered better—that is, untrustworthy faces played a more important role in the formation of first impressions.

Based on the EASI model, we found that only the effect of facial expression on the trustworthiness judgments was affected by context. Van Kleef (2009) argued that people are more likely to use emotional responses to process emotional information in a context of collaboration and are more likely to use the inference pathway in a competitive environment. Our research, to some extent, supports the above argument. We found that the pathway of emotional response plays a role in both types of settings; however, the emotional face effect was smaller in a competitive setting than in a cooperative setting. Specifically, in a cooperative setting, individuals utilize the emotional response pathway to process emotional information, and emotional information of the expresser directly stimulates the same emotional state in the observer, leading to trust effects at the emotional level. In this context, other people’s facial expression has a “top-down” start-up impact on the observer (Van Kleef et al., 2010a). Therefore, a happy facial expression increases the observer’s trust in the observe, while an angry expression does the opposite. However, in a competitive setting, it is not easy to accurately grasp the emotional information of others because of the complexity of the social environment, and the inference processing pathway plays a more significant role (Van Kleef et al., 2010a). Therefore, while facial expression still plays a pivotal role in competitive settings, the emotional face effect was smaller than in cooperative settings. Our experimental results further confirm the validity of the dual processing pathways.

Furthermore, it is worth noting that when the facial expression becomes angry, the participants did not show a heightened angry expression effect in the competitive setting than in cooperative setting. As proposed by the EASI model (Van Kleef et al., 2010b), this may be because the expression of anger is likely to trigger competition in cooperative settings and cooperation in competitive settings. In fact, angry faces indicate refusal and rejection (Heerdink et al., 2014), which might trigger an instinct to leave, however, observers cannot leave cooperative settings (observee and observer are mutually interdependent). Therefore, the observer can only select a reduction of trust in that condition. However, observers may feel highly pressured in competitive settings, so they may give in to the observee’s desires so as to prevent more negative consequences (Van Kleef et al., 2010b).

Thus, our experiments prove that in trustworthiness judgments, facial expression is affected by context and that the emotional response pathway is dominant in trustworthiness judgments of facial expression, while inference processing also plays a regulatory role in different situations. Facial gender presents cross-setting stability. Therefore, facial gender has a more stable effect on trustworthiness judgments than facial expression in social context. However, the EASI model suggests that the processing approach of the emotional response pathway acts by stimulating the same emotional state of the expresser in the observer. Thus, future research should consider observers’ emotional state as well as diverse social contexts, emotional intensity, cultural factors, and the gender of the rater (Debruine, 2005).

## Data Availability Statement

The raw data supporting the conclusions of this manuscript will be made available by the authors, without undue reservation, to any qualified researcher.

## Ethics Statement

This study was carried out in accordance with the recommendations of the Research Ethics Committee of Renmin University of China with written informed consent from all subjects. All participants gave written informed consent in accordance with the Declaration of Helsinki. The protocol was approved by the Research Ethics Committee of Renmin University of China.

## Author Contributions

YD and YJ designed the research. YJ collected the data. YJ, YiL, and CL analyzed the data. YD, YiL, YoL, and CL discussed the current results based on the previous literature. YD, YJ, YiL, and YoL wrote the manuscript. All the authors read and approved the final manuscript.

## Conflict of Interest Statement

The authors declare that the research was conducted in the absence of any commercial or financial relationships that could be construed as a potential conflict of interest.
